# Application of Carotid Duplex Ultrasonography in the Surveillance of Carotid Artery Stenosis after Neck Irradiation

**DOI:** 10.31083/j.rcm2307240

**Published:** 2022-06-27

**Authors:** Deng-Yu Kuo, Yen-Wen Wu, Chen-Hsi Hsieh, Li-Jen Liao, Pei-Wei Shueng

**Affiliations:** ^1^Division of Radiation Oncology, Department of Radiology, Far Eastern Memorial Hospital, 220 New Taipei, Taiwan; ^2^Division of Cardiology, Cardiovascular Medical Center, Far Eastern Memorial Hospital, 220 New Taipei, Taiwan; ^3^School of Medicine, College of Medicine, National Yang Ming Chiao Tung University, 112 Taipei, Taiwan; ^4^Institute of Traditional Medicine, National Yang Ming Chiao Tung University, 112 Taipei, Taiwan; ^5^Department of Otolaryngology Head and Neck Surgery, Far Eastern Memorial Hospital, 220 New Taipei, Taiwan; ^6^Biomedical Engineering Office, Far Eastern Memorial Hospital, 220 New Taipei, Taiwan; ^7^Department of Electrical Engineering, Yuan Ze University, 320 Taoyuan, Taiwan; ^8^Medical Device Innovation and Translation Center, National Yang Ming Chiao Tung University, 112 Taipei, Taiwan

**Keywords:** carotid artery stenosis, radiotherapy, head and neck cancer

## Abstract

Head and neck cancer (HNC) shares some risk factors with cardiovascular disease. Neck radiotherapy (RT) causes carotid artery injury and stenosis. In HNC patients treated with RT, the prevalence rate of severe (>70%) carotid artery stenosis is >10%, and the cumulative incidence continuously increases over time. There is at least a two-fold risk of cerebrovascular events in these patients compared with the normal population. Carotid artery stenosis is mainly assessed and diagnosed via duplex ultrasonography. Angioplasty and stenting may be recommended to patients who developed severe post-irradiation carotid artery stenosis. This review assessed Taiwanese data that provided some recommendations for HNC patients treated with RT. With consideration of the high prevalence rate of carotid artery stenosis after neck irradiation, duplex ultrasonography should be included in the follow-up workup.

## 1. Introduction 

Head and neck cancer (HNC) accounts for approximately 900,000 new cases and 400,000 deaths annually worldwide [1]. Squamous cell carcinoma is the most common histology of HNC, and it usually develops in the mucosal surfaces of the oral cavity, pharynx, and larynx. Tumors originating from the salivary gland, nasal cavity, or paranasal sinus are less frequently observed. Patients with HNC are at high risk of neck nodal metastasis because the neck is rich in lymphatic drainage. Thus, neck irradiation is indicated in more than half of patients with HNC. For patients with oral cavity cancer who have adverse risk factors, such as close margin or perineural invasion, postoperative radiotherapy (RT) can be beneficial for locoregional control. To preserve organs or manage unresectable HNC tumors, definitive RT is the mainstay local treatment [2]. After diagnosis and treatment, the primary HNC accounted for 73.4% of deaths within the first 5 years. However, death from competing causes became more common [3]. Nearly one in three patients with HNC die from competing etiologies, and the most common causes include cardiovascular disease (CVD) and lung cancer and other types [4, 5].

HNC and CVD have similar risk factors such as male sex, low fruit and vegetable intake, and tobacco and alcohol use [6]. In HNC patients, the prevalence rates of carotid artery disease and any CVD, which can possibly be underestimated, are 3% and 26%, respectively [7]. Cardio-oncology, a multidisciplinary approach for the detection, monitoring, and treatment of cardiovascular dysfunction in patients with cancer, has been an important issue in recent years [8, 9, 10]. That is, anthracycline and trastuzumab for breast cancer, androgen deprivation therapy for prostate cancer, and heart radiation dose for esophageal or breast cancer are associated with CVD [9, 11, 12, 13, 14]. In treating HNC, the RT dose is up to 70 Gy, which can damage the carotid artery leading to stenosis. Carotid artery stenosis does not cause significant symptoms if the lumen narrowing is <70%. Transient ischemic attack (TIA) or ischemic stroke may suddenly occur, and it can be attributed to severe neurologic sequelae. Patients with carotid bruits upon neck auscultation are at high risk of cerebrovascular events [15]. The current guidelines do not recommend screening for bruits during physical examination as it has poor reliability and sensitivity. Instead, duplex ultrasonography has adequate evidence for detecting carotid artery stenosis [16].

Although there are published reviews and consensus reports focusing on the cardio-oncology, post-irradiated carotid artery stenosis in HNC is not commonly discussed [17, 18, 19, 20]. Due to improvement in long-term HNC tumor control, radiation-related late toxicity is increasingly considered. The current review aimed to comprehensively assess existing data about the prevalence, pathogenesis, risk factors, diagnosis, and treatment of post-irradiation artery stenosis. In addition, we collected Taiwanese data and information about future perspectives. With consideration of the high prevalence rate of carotid artery stenosis after neck irradiation in HNC patients, duplex ultrasonography should be included in the follow-up workup.

## 2. Prevalence of Carotid Artery Stenosis

In the general population, the prevalence rates of asymptomatic moderate (>50%) and severe (>70%) carotid artery stenoses are 4.2% and 1.7%, respectively, and these increase with age and male sex [21]. In addition, the carotid intima-media thickness (CIMT) and local arterial stiffness are positively correlated with the serum uric acid level [22, 23]. Considering the prevalence of post-irradiation carotid stenosis in patients with HNC, Table 1 (Ref. [24, 25, 26, 27, 28, 29, 30, 31, 32, 33]) shows the results of selected studies that used different approaches. Researchers commonly screened the carotid artery for asymptomatic patients via ultrasonography and calculated the prevalence of stenosis. Cheng *et al*. [24] performed a comparative cross-sectional study to investigate the prevalence of radiation-induced carotid stenosis. In total, 240 patients who received neck irradiation, with a mean interval of 72 months, were included in the study. In 28 (11.7%) patients, >70% carotid artery stenosis was detected via color flow duplex scan. Carpenter *et al*. [28] retrospectively included 366 patients with HNC undergoing carotid ultrasonography screening after neck irradiation. Carotid artery stenosis was defined as ≥50% stenosis, stroke, or TIA. The 2-, 5-, and 8-year cumulative incidence rates of composite carotid stenosis were 11%, 20%, and 29%, respectively. A meta-analysis enrolled 1928 patients in 12 studies focusing on patients with nasopharyngeal carcinoma (NPC) treated with RT. Patients who received RT had a higher incidence of overall stenosis (risk ratio = 4.17) and significant (≥50%) stenosis (risk ratio = 8.72) [29]. Another meta-analysis including 19 studies with 1479 patients showed that the prevalence rates of >50% and >70% carotid stenosis and carotid occlusion after irradiation were 25%, 12%, and 4%, respectively. Meanwhile, the 12-, 24-, and 36-month cumulative incidence of >50% carotid stenosis were 4%, 12%, and 21%, respectively [30].

**Table 1.** **Prevalence of post-RT carotid artery stenosis based on different study types**.

First author/Reference no.		Year	Cancer	RT group	Non-RT group	Median FUI (years)	Endpoints	Results
Cross-sectional								
	Cheng [24]	1999	HN	240	-	6	>70% stenosis	11.70%
	${\mathrm{Chang}}^{\text{a}}$ [25]	2009	HN	192	98	2	>50% stenosis	19.8% vs 0%
Retrospective cohort								
	Dorresteijn [26]	2002	HN	367	-	7.7	Cumulative risk of stroke	15-year 12%
	Haynes [27]	2002	HN	413	-	Not mentioned	Rate of stroke	5-year 12%
	Carpenter [28]	2018	HN	366	-	4.1	≥50% stenosis, stroke, or TIA	2-year 11%, 5-year 20%, 8-year 29%
Meta-analysis								
	Liao [29]	2019	NPX	837 (12 studies)	1091	4–14	Risk ratio of overall, ≥50% stenosis	4.71, 8.72
	Texakalidis [30]	2020	HN	1479 (19 studies)	-	2–13	>50%, >70%, total stenosis	25%, 12%, 4%
							>50% stenosis	1-year 4%, 2-year 12%, 3-year 21%
Database cohort								
	Smith [31]	2008	HN	1983 (RT alone),	2056 (surgery alone)	2.4	10-year cerebrovascular events	34% vs 25% vs 26%
				2823 (surgery + RT)				
	${\mathrm{Huang}}^{\text{a}}$ [32]	2011	HN	4391 (RT or CT), 2880 (surgery + adjuvant)	2901 (surgery alone)	5.8	Stroke	3.8% vs 3.2% vs 4.3%
	${\mathrm{Wu}}^{\text{a}}$ [33]	2015	Oral	11,905 (RT or CT), 3967 (surgery + adjuvant)	5981 (surgery alone)	Not mentioned	Ischemic stroke	7.4% vs 6.1% vs 6.5%

Footnotes: ^a^ Taiwanese study. Abbreviations: HN, head and neck; NPX, nasopharynx; RT, radiotherapy; CT, chemotherapy; FUI, follow-up interval; TIA, transient ischemic attack.

Another approach is to use cerebrovascular events as the end point. Higher risk of stroke in patients treated with neck RT implies that the radiation causes the development of carotid artery stenosis. Dorresteijn *et al*. [26] followed-up 367 patients with HNC who received RT before the age of 60 years. Results showed that the 15-year cumulative risk of stroke was 12.0%. Haynes *et al*. [27] retrospectively evaluated 413 patients with HNC treated with neck irradiation. In total, 20 patients had stroke (crude incidence of 4.8%) in the follow-up period, ranging from 2 and 146 months. The 5-year actuarial rate of stroke was 12%, and the relative risk was 2.09 compared with expected data. Smith *et al*. [31] identified 6862 patients who were aged >65 years and diagnosed with nonmetastatic HNC from the Surveillance, Epidemiology, and End Results cohort. The 10-year incidence of cerebrovascular events (stroke, carotid revascularization, or stroke-related death) was significantly higher in patients treated with RT alone than those managed with surgery (34% vs 26%, *p *< 0.001). However, such difference was not observed in the surgery plus RT group (10-year incidence of 25%). These results can be attributed to a higher radiation dose in the RT alone group than in the adjuvant RT group. A comprehensive review addressed the risk of ischemic stroke and TIA after head and neck irradiation. There were 17 trials investigating the epidemiology. Results showed that RT at least doubled the relative risk of cerebrovascular events in the different follow-up periods [34].

## 3. Pathogenesis

**Fig. 1.**
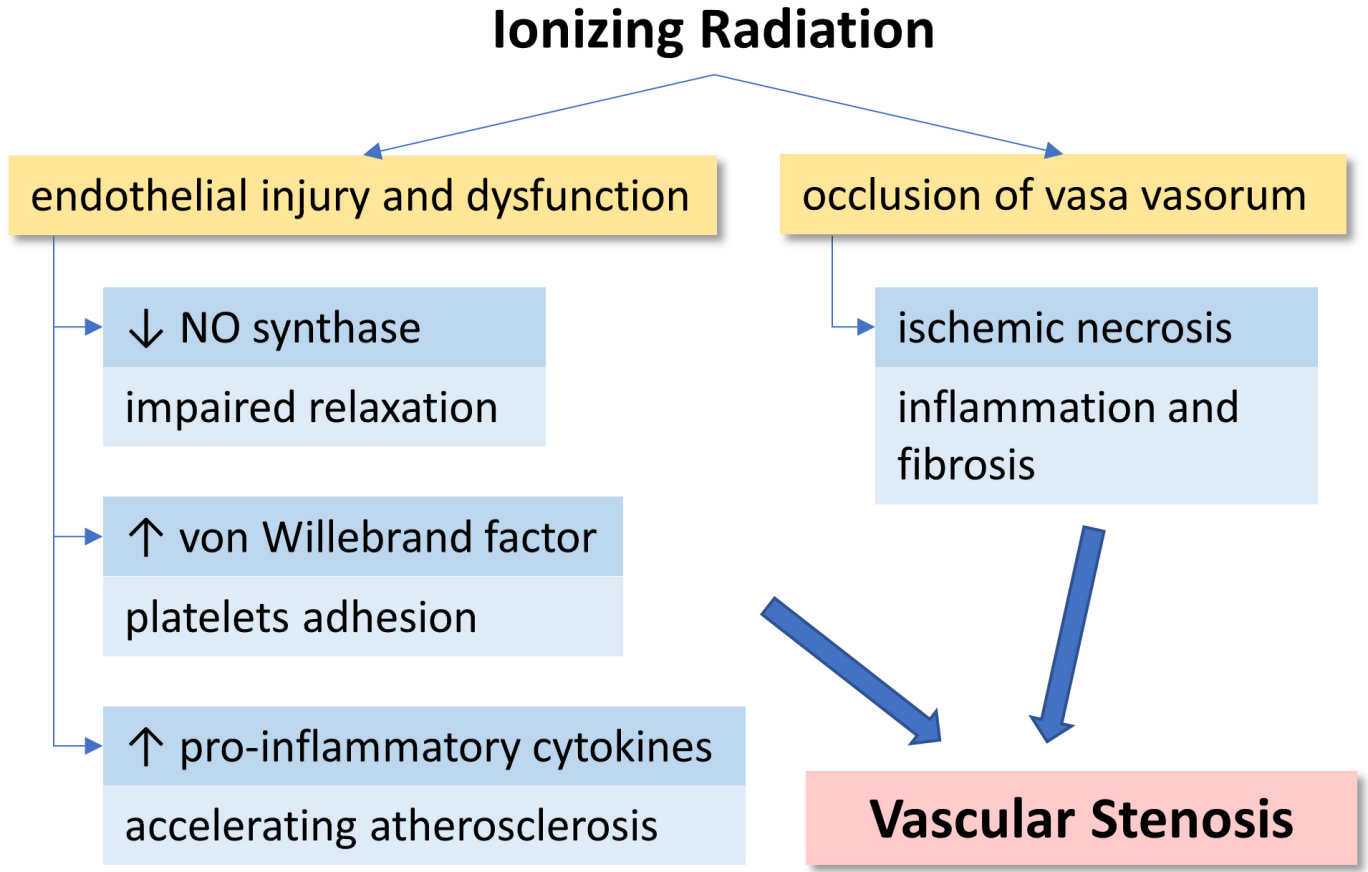
**Pathogenesis of radiation-induced vascular stenosis**. Two major mechanisms, endothelial dysfunction and occlusion of vasa vasorum contributed to the result.

The pathogenesis of radiation-induced vascular disease is not fully elucidated. However, it is likely multifactorial (Fig. 1). Some of the mechanisms are endothelial injury and dysfunction, which are characterized by impaired endothelium-dependent relaxation with a lack of nitric oxide synthase expression [35, 36, 37]. Moreover, radiation induces endothelial cells to release von Willebrand factor, which enhances platelet adhesion and predisposes to arterial thrombosis [38]. Radiation, even at low doses, induces the release of pro-inflammatory cytokines (such as interleukin [IL]-1, IL-6, tumor necrosis factor alpha, and tumor growth factor beta [TGF-β]), which are associated with accelerating atherosclerosis [36, 39]. Some authors showed that the occlusion of vasa vasorum causing ischemic necrosis is the predominant mechanism of radiation-induced vascular disease [40, 41]. The elastic tissues and muscle fibers are then replaced by fibrotic tissues, which cause increased intima-media thickness [42]. An animal study described acute and chronic morphologic changes after 40-Gy irradiation for 10 days [43]. Within 48 h, the endothelium showed moderate to severe acute injury. New cells repopulated, and the luminal surface thickened within 3 weeks. By contrast, progressive inflammation and fibrosis were noted in the media and adventitia layers. The latter morphologic alterations were long-lasting.

There are several hypotheses for the development of classic atherosclerosis, including ‘response to injury’, ‘oxidized low density lipoprotein’, and ‘inflammation’. Atherosclerosis is associated with many risk factors, such as obesity, hypercholesterolemia, hypertension, diabetes, and smoking [44, 45]. On the other hand, some characteristics of radiation-induced carotid artery stenosis differed from those of classic atherosclerosis. Patients with post-RT carotid artery stenosis had fewer atherosclerotic risk factors. In human pathological studies, medial thinning and adventitia fibrosis were observed in the vessels after irradiation [46]. Less inflammatory, more fibrotic, and a smaller lipid core size were associated with radiation-induced plaque [47]. Lam *et al*. [48] investigated the distribution of plaques, and results showed that the most often affected sites were the common carotid arteries in patients treated with irradiation, while the carotid bulb in controls. Post-irradiated stenotic lesions might more likely affect the carotid artery diffusely and occur bilaterally, and maximal stenosis could commonly develop at the end of the stenotic area [49, 50].

## 4. Radiation Dose and Interval after Irradiation

Neck irradiation can induce carotid artery stenosis. Even with moderate-dose RT, atherosclerosis may develop [51]. For example, patients treated for Hodgkin lymphoma with 40-Gy irradiation had a higher incidence of TIA or stroke in the long-term follow-up, with a risk ratio of approximately 5 [52, 53]. Patients with HNC can receive a higher neck radiation dose, ranging from 50 to 70 Gy, depending on the definitive or adjuvant setting. A positive correlation was noted between dose and atherosclerotic activity [25].

However, the dose-effect relationship, which may be confounded with other risk factors and follow-up interval, remains unclear. Dorth *et al*. [54] retrospectively reviewed patients with HNC who underwent carotid ultrasonography. They found an insignificant dose-effect that might be correlated with carotid artery stenosis with every 10 Gy increase in mean RT dose, with a hazard ratio of 1.4. A study included patients who received ipsilateral neck RT and compared the prevalence of carotid stenosis and intima-media thickness on each side [55]. Results found increased intima-media thickness and a higher grade of carotid bulb abnormality at a dose of >35 Gy. However, this study only had 40 cases and included a large proportion of patients with lymphoma, which cannot be extrapolated directly to patients with HNC.

Moreover, the interval after RT plays an important role in the development of carotid stenosis. Cheng *et al*. [24] revealed that time interval from RT is an independent predictor of severe carotid artery stenosis via a multivariate logistic regression analysis. Dorresteijn *et al*. [26] compared the risk of ischemic stroke between patients receiving neck irradiation and normal population. After adjusting for sex, age, and other risk factors for stroke, the relative risk was 3.7 within the 10-year follow-up, which increased to 10.1 after >10 years. As previously mentioned, radiation-induced vascular injury may evolve into a long-term sequela.

Although the pathogenesis varies, radiation-induced carotid artery stenosis cannot be totally spared from classic atherosclerosis. The risk factors of patients with ischemic stroke include hypertension, dyslipidemia, diabetes mellitus, obesity, smoking, and alcohol consumption [56]. A meta-analysis included prospective or retrospective observational studies reviewing patients with a history of head and neck RT. A research compared the baseline characteristics between patients with stenosis and those without. Results showed that diabetes and smoking could be the possible risk factors of severe carotid stenosis, with odds ratios of 3.67 and 4.48, respectively. However, no such significant difference was noted in terms of the incidence of hypertension and coronary artery disease [30].

## 5. Diagnostic Tools

Asymptomatic carotid artery stenosis is commonly diagnosed via duplex ultrasonography, which comprises the Doppler and B-modes. Doppler ultrasonography can evaluate the velocity and direction of the blood flow using a color scale. The B-mode shows two-dimensional images with a grayscale, which provides information about the plaque features and thickness of the arterial wall. The severity of stenosis depends on the peak systolic velocity and the presence of plaques. Other criteria, such as collateral flow, prestenotic flow, and poststenotic flow disturbances, can increase the reliability of results [57]. The European Society of Cardiology recommends ultrasonography as the first-line examination, and a threshold of 70% stenosis was set for the indication of revascularization [58].

The development of carotid artery stenosis may take years. However, the assessment of CIMT is useful in predicting cardiovascular diseases [59]. Using high-resolution B-mode ultrasonography, the vascular structure can be visualized. CIMT is evaluated using a longitudinal image of the carotid artery with a double-line pattern, which comprises the lumen–intima and media–adventitia interfaces. Previous studies have shown that radiation increases the CIMT via a dose-effect [60, 61]. In patients treated with neck irradiation, duplex ultrasonography and CIMT assessment are useful for the early detection of carotid artery stenosis.

Digital subtraction angiography is the gold standard for diagnosing vascular stenosis. However, the procedure is invasive and time-consuming. Noninvasive imaging modalities, such as computed tomography angiography (CTA) and magnetic resonance angiography (MRA), have evolved, and they replaced the diagnostic role of digital subtraction angiography and became a complement of ultrasonography [58]. CTA provides three-dimensional images with a better spatial resolution than duplex ultrasonography. The sensitivity and specificity of detecting severe carotid artery stenosis via dual-source CTA are >95% [62, 63]. MRA had comparable results without ionizing irradiation [62, 63, 64, 65]. The applications of CTA and contrast-enhanced MRA are restricted in patients with impaired renal function. Although with less accuracy, non-contrast-enhanced MRA is an alternative in some cases [62, 66].

## 6. Treatment

Although the pathogenesis of radiation-induced carotid artery stenosis differs from that of classical atherosclerosis, lifestyle modification (e.g., weight control and smoking cessation) and risk factors (e.g., hypertension, diabetes mellitus, dyslipidemia) control are still important for preventing TIA and ischemic stroke [56]. Currently, there is no large clinical trial investigating medical treatment specifically for radiation-induced carotid stenosis. Based on *in vitro* studies, statins had anti-inflammatory and anti-thrombotic effects on irradiated endothelial cells, which may be considered in therapeutic strategies [67]. A retrospective cohort study showed that the use of statins after RT was associated with a significant reduction in the incidence of stroke, with a hazard ratio of 0.68 [68]. In addition, the use of perioperative statins can reduce the incidence of cerebrovascular events and mortality among patients undergoing carotid endarterectomy (CEA) [69]. Other medications, such as angiotensin-converting enzyme inhibitors and antiplatelet drugs, may have benefits [58].

In some cases, intervention is indicated for symptomatic patients. CEA is an invasive surgery for removing plaques from the carotid artery to improve blood flow. Randomized trials have shown the benefit of CEA among symptomatic patients with high-grade (>70%) atherosclerotic carotid stenosis, with 16% reduction in the absolute risk of ipsilateral ischemic stroke within 5 years. In contrast, for patients with <50% stenosis, CEA did not have significant benefits [56, 70]. The 30-day stroke or death rate after this procedure was 7.1% [70]. Carotid artery angioplasty and stenting (CAS) is a less invasive percutaneous procedure that can be used as an alternative to CEA. After a successful CAS in patients with severe carotid artery stenosis, recovery of cerebral perfusion and improvement in neurocognitive function could be observed [71, 72]. The Cochrane Group summarized 22 randomized trials comparing the efficacy of CAS and CEA [73]. In patients with standard surgical risk, the primary outcome did not differ during the follow-up period. There was a trend of increasing incidence of periprocedural complications with stenting. The selection of revascularization procedure may be influenced by anatomy, prior illness or treatment, and patient risk [58]. After irradiation, poor circulation and tissue fibrosis could increase surgical difficulties and complication risks. A pooled analysis of 27 articles comprising 533 patients undergoing previous neck irradiation and carotid artery revascularization showed that both CAS and CEA were feasible with a low risk of cerebrovascular adverse events. However, CEA can more likely cause temporary cranial nerve injuries, and CAS was associated with higher rates of restenosis [74]. In patients at high risk for surgery such as post neck irradiation, the American Heart Association guidelines recommend CAS [56].

## 7. Taiwanese Data

Cancer is the leading cause of death in Taiwan. The incidence of head and neck (oral cavity, oropharynx, and hypopharynx) squamous cell cancers is relatively high, with a crude rate of 34 per 100,000 people in 2018. There were 8170 newly diagnosed cases and 3027 deaths, accounting for 7% of new cancer cases and 6.2% of all cancer-related deaths [75]. HNC originated from the oral cavity are in majority and associated with the consumption of cigarette, alcohol, and betel quid [76]. Although the incidence of human papillomavirus (HPV)-associated oropharyngeal cancer is increasing, the HPV-positivity rate is still <30% in Taiwan [77]. NPC is more prevalent in Taiwan than in western countries. More than 1400 patients with NPC are diagnosed annually, accounting for 1.3% of all new cancer cases [75]. The epidemiology of HNC in Taiwan is quite different from that in other countries. Thus, local research data are important for improving daily clinical practice. Taiwanese studies focusing on radiation-induced carotid stenosis were conducted in this review.

In some institutions, duplex ultrasonography is performed regularly to assess the patency of carotid arteries among patients treated with neck irradiation. Chang *et al*. [25] conducted a prospective, cross-sectional study to evaluate the prevalence of radiation-induced carotid artery stenosis via carotid duplex sonography. In total, 290 consecutive patients with HNC were enrolled. With a median 2-year interval after RT, the incidence rates of >50% and >70% carotid stenosis were 19.8% and 8.9%, respectively. In the control group without RT, none of the patients had >50% carotid stenosis. In addition, the plaque score of the RT group was significantly higher than that of the non-irradiated group. Liu *et al*. [78] performed annual carotid duplex ultrasonography after RT on stroke-naïve patients with HNC to monitor carotid artery stenosis progression. The total plaque score (TPS) was defined as the sum of five segment grades (1 to 5) including the proximal common carotid artery, distal common carotid artery, carotid bifurcation, internal carotid artery, and external carotid artery. Patients with a TPS of ≥7 had a higher risk of carotid artery stenosis and ischemic stroke. Liu *et al*. [79] also found the prevalence of post-RT hypothyroidism might increase to 50% with time. They hypothesized that hypothyroidism may raise atherogenic risks and assessed the association between post-RT hypothyroidism and carotid artery stenosis. Patients with HNC treated with RT were categorized into the euthyroid and hypothyroidism groups. TPS and degrees of carotid artery stenosis were assessed annually. However, there was no significant difference in terms of carotid artery stenosis progression and ischemic stroke incidence between the two groups. Yeh *et al*. [80] assessed the CIMT, a strong predictor for CVD, in 70 patients. Results showed that neck irradiation is the most important risk factor in patients with a CIMT of ≥1.0 mm, with an odds ratio of 13.5 in the multivariate analysis. The mean CIMTs were 0.82 and 0.58 mm in patients who received prior neck RT and those without cancer, respectively. In patients with NPC, RT is the mainstay treatment and is associated with a good control rate for locoregional disease. The post-irradiation carotid artery stenosis could be evaluated without the effect of neck dissection. Huang *et al*. [81] evaluated 105 patients with NPC who were at 1-year post-RT and 25 healthy control subjects via B-mode ultrasonography. The NPC group had a significantly greater CIMT than the control group (1.0 vs 0.6 mm, *p *< 0.001). Approximately 36% of patients with NPC presented with carotid plaque, which was associated with age and duration after RT, with a cutoff value of 52.5 years and 42.5 months, respectively.

Other studies used data from different databases to assess the correlation between neck irradiation and ischemic stroke. Li *et al*. [82] conducted a case-control study including 319 patients with ischemic stroke with or without NPC after neck irradiation. Patients treated with RT were younger but with higher proportions of carotid artery disease (42% vs 11%; *p *< 0.0001). Results showed that neck irradiation may cause accelerated atherosclerosis independent from other risk factors. A cohort study comprised 10,172 patients with HNC from the National Health Insurance Research Database (NHIRD) [32]. At a median follow-up of 5.8 years, 384 patients had stroke. Patients aged <55 years who received RT or chemotherapy had a 1.8-fold higher risk of stroke compared with those who underwent surgery alone. However, such difference was not significant among older patients. Another study collected the data of 21,853 patients with oral cancer from the NHIRD [33]. The risk of ischemic stroke was greater in patients treated with RT or chemotherapy than those who underwent surgery alone (hazard ratio = 1.24). The incidence of ischemic stroke increased with age. However, the age-specific hazard ratio was greater in younger patients. Based on the two abovementioned studies, younger patients have less atherosclerotic risk factors, such as hypertension and hyperlipidemia. Thus, RT accounts for a larger proportion of ischemic stroke in this group than the elderly.

Considering the treatment of carotid artery stenosis, CAS, rather than CEA, is indicated for patients with severe radiation-associated carotid stenosis. Huang *et al*. [83] reported the long-term outcomes of carotid artery stenting. The procedures were performed on 129 patients with a mean follow-up of 42.7 months. Between the radiation and non-irradiated groups, there were no significant differences in primary end points, including 30-day major complications and 5-year freedom from mortality, ipsilateral recurrent stroke, and major adverse cardiovascular events. Results showed that the outcomes of carotid artery stenting did not change based on the history of neck irradiation, except for asymptomatic carotid restenosis.

## 8. Future Perspectives

The best method for preventing radiation-induced carotid artery stenosis is lowering the radiation dose as much as possible. Precision and personalized medicine play an important role in cancer treatment. The radiation dose could be reduced or omitted in some cases. For example, patients with NPC commonly present with bilateral cervical lymph node metastases. Thus, prophylactic whole neck irradiation is indicated. However, recent studies showed that selective neck irradiation with a lower elective dose is feasible among patients with NPC [84, 85]. In HPV-positive oropharyngeal cancer, phase II trials revealed that dose de-escalation had a comparable locoregional tumor control with less toxicity [86, 87]. Proton beam, with physical advantage of Bragg peak, can achieve a rapid fall-off of the radiation dose to the surrounding organ. A pilot study showed that intensity-modulated proton therapy could reduce the dose to the vertebral artery in the NPC treatment plan [88]. Carotid artery sparing could be achieved in early-stage laryngeal cancer [89]. Proton therapy can reduce the dose at the carotid artery, particularly in the sequence of gross tumor boost without prophylactic neck irradiation.

Early detection is important for identifying asymptomatic patients who presented with post-irradiation carotid artery stenosis. Although duplex ultrasonography and CIMT are good screening tools, some novel biomarkers are still under investigation. Because inflammation is a key process in atherosclerosis, the association between CVD and several inflammatory markers was assessed [90]. For example, a study showed that high-sensitivity C-reactive protein was an independent predictor of future cardiovascular events [91]. Similarly, fluorodeoxyglucose (FDG)-positron emission tomography scan is also a useful tool for detecting carotid plaque inflammation, which is a marker of symptomatic carotid artery disease [92]. Chen *et al*. [93] conducted a pilot study including patients with HNC treated with chemoradiation and arranged pre- and 3 months post-RT. Results showed a significantly higher FDG uptake in the carotid artery, which can be an early biomarker of radiation-induced vascular injury.

Previously, radiation-induced fibrosis was believed to be an inevitable and irreversible process. However, new treatments involving the pathway of radiation-induced fibrosis have been assessed. For example, TGF-β, a pro-inflammatory cytokine, can trigger fibroblasts and induce late fibrosis. Animal studies showed that the use of IPW-5371, a small molecule TGF-β receptor 1 inhibitor, is an effective radiation countermeasure as it reduces fibrosis [94, 95]. Moreover, mesenchymal stem cells have tissue regeneration, strong immunomodulation, and anti-inflammatory activities [96]. These cells could migrate to the site of vessel injury and differentiate into endothelial cells [97, 98]. Hence, it is a promising research topic for the prevention or treatment of post-irradiated carotid artery stenosis.

## 9. Conclusions

In summary, in patients with HNC treated with RT, the prevalence of severe carotid artery stenosis is >10%, and the cumulative incidence continuously increases over time, which leads to at least a two-fold risk of cerebrovascular events. The clinical practice guidelines for HNC have recommended the monitoring of late RT toxicities, including assessment of thyroid function, dental evaluation, and speech/swallowing rehabilitation [2, 99]. However, the screening or follow-up of post-irradiated carotid artery stenosis has not been discussed.

**Fig. 2.**
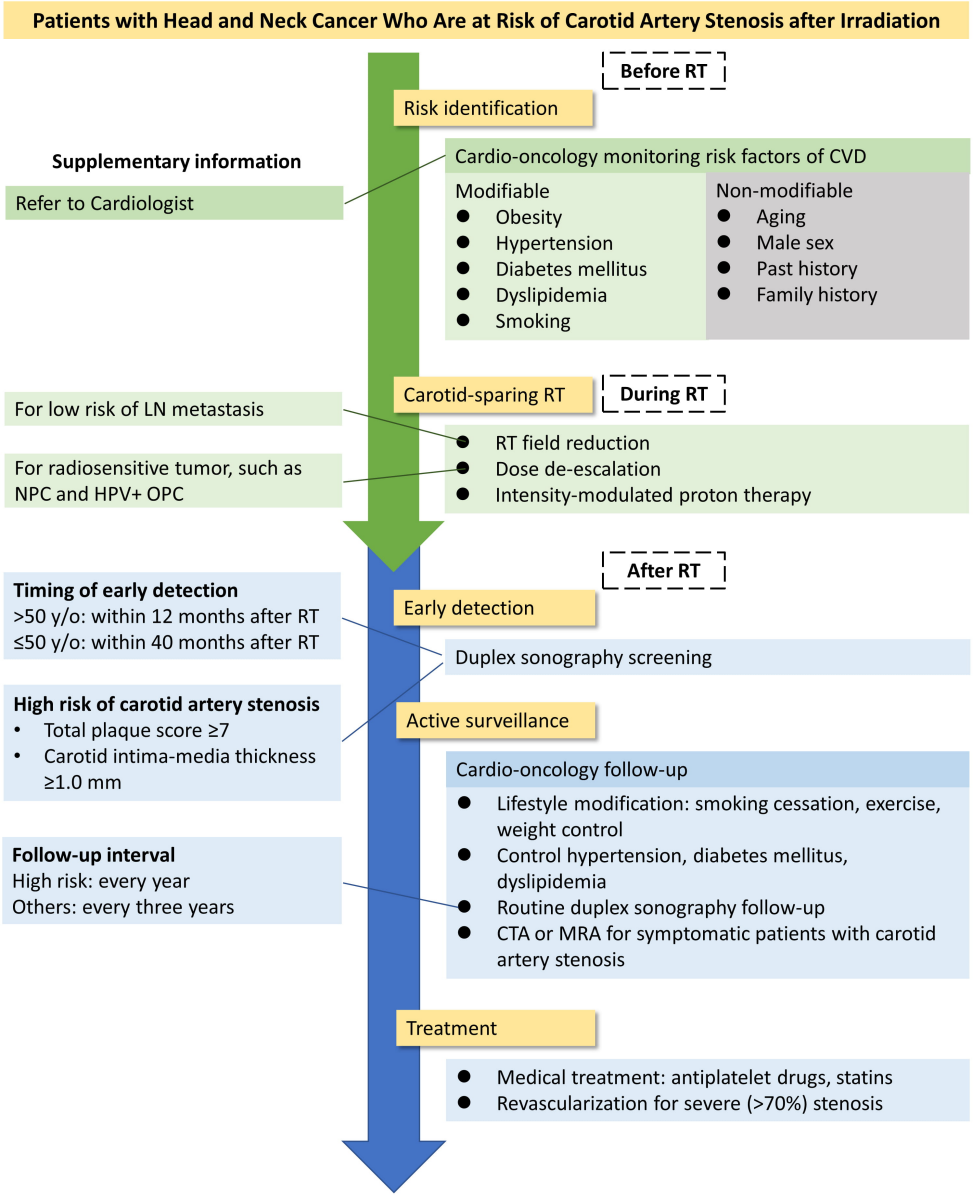
**Approach for patients with head and neck cancer treated with radiotherapy**. Abbreviations: CTA, computed tomography angiography; CVD, cardiovascular disease; HPV+OPC, human papillomavirus positive oropharyngeal cancer; LN, lymph node; NPC, nasopharyngeal cancer; MRA, magnetic resonance angiography; RT, radiotherapy.

Fig. 2 shows the possible approach for patients with HNC receiving neck irradiation. Clinicians should identify and control the risk factors associated with carotid artery stenosis. Personalized RT planning design could reduce the carotid artery dose. Screening is essential for detecting symptom-free carotid artery stenosis that causes unexpected disability or death. Patients who are aged >50 years and those who are at 40 months post-RT are at higher risk for carotid artery stenosis and should undergo duplex ultrasonography. In patients with a TPS of ≥7 or a CIMT of ≥1.0 mm, close monitoring and proper treatment should be considered.
